# 2-Chloro-6-methyl­quinoline-3-carbaldehyde

**DOI:** 10.1107/S1600536809040653

**Published:** 2009-10-10

**Authors:** F. Nawaz Khan, R. Subashini, S. Mohana Roopan, Venkatesha R. Hathwar, Seik Weng Ng

**Affiliations:** a Chemistry Division, School of Science and Humanities, VIT University, Vellore 632 014, Tamil Nadu, India; b Solid State and Structural Chemistry Unit, Indian Institute of Science, Bangalore 560 012, Karnataka, India; c Department of Chemistry, University of Malaya, 50603 Kuala Lumpur, Malaysia

## Abstract

The quinolinyl fused-ring of the title compound, C_11_H_8_ClNO, is almost planar (r.m.s. deviation = 0.013 Å); the formyl group is slightly bent out of the plane of the fused ring system [C—C—C—O torsion angle = 13.5 (4)°].

## Related literature

For a review of the synthesis of quinolines by the Vilsmeier–Haack reaction, see: Meth-Cohn (1993 ▶).
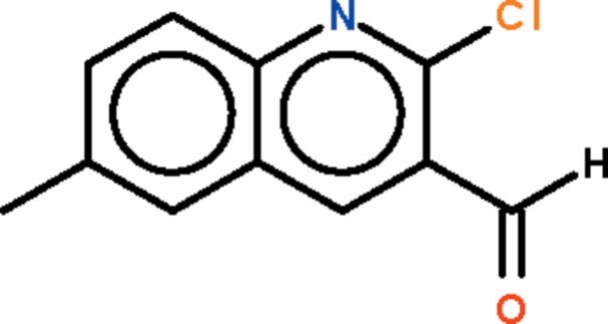


## Experimental

### 

#### Crystal data

C_11_H_8_ClNO
*M* *_r_* = 205.63
Monoclinic, 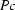

*a* = 5.944 (1) Å
*b* = 3.9210 (19) Å
*c* = 20.390 (2) Å
β = 101.377 (15)°
*V* = 465.9 (2) Å^3^
*Z* = 2
Mo *K*α radiation
μ = 0.37 mm^−1^
*T* = 290 K
0.25 × 0.15 × 0.15 mm

#### Data collection

Oxford Diffraction Excalibur diffractometer
Absorption correction: multi-scan (*CrysAlis Pro*; Oxford Diffraction, 2009 ▶) *T*_min_ = 0.913, *T*_max_ = 0.947
5980 measured reflections
2052 independent reflections
1831 reflections with *I* > 2σ(*I*)
*R*_int_ = 0.028

#### Refinement

*R*[*F* ^2^ > 2σ(*F* ^2^)] = 0.034
*wR*(*F* ^2^) = 0.089
*S* = 1.00
2052 reflections
128 parameters
2 restraints
H-atom parameters constrained
Δρ_max_ = 0.19 e Å^−3^
Δρ_min_ = −0.17 e Å^−3^
Absolute structure: Flack (1983 ▶), 990 Friedel pairs
Flack parameter: 0.02 (6)

### 

Data collection: *CrysAlis Pro* (Oxford Diffraction, 2009 ▶); cell refinement: *CrysAlis Pro*; data reduction: *CrysAlis Pro*; program(s) used to solve structure: *SHELXS97* (Sheldrick, 2008 ▶); program(s) used to refine structure: *SHELXL97* (Sheldrick, 2008 ▶); molecular graphics: *X-SEED* (Barbour, 2001 ▶); software used to prepare material for publication: *publCIF* (Westrip, 2009 ▶).

## Supplementary Material

Crystal structure: contains datablocks global, I. DOI: 10.1107/S1600536809040653/tk2550sup1.cif

Structure factors: contains datablocks I. DOI: 10.1107/S1600536809040653/tk2550Isup2.hkl

Additional supplementary materials:  crystallographic information; 3D view; checkCIF report

## Figures and Tables

**Fig. 1.**
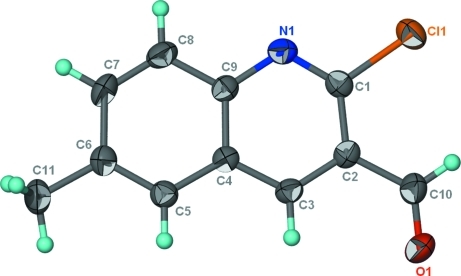
Thermal ellipsoid plot (Barbour, 2001) of C11H8ClNO at the 50% probability level; hydrogen atoms are drawn as spheres of arbitrary radius.

## supplementary crystallographic information

### Experimental

A Vilsmeier-Haack adduct prepared from phosphorus oxytrichloride (6.5 ml, 70 mmol) and *N*,*N*-dimethylformamide (2.3 ml, 30 mmol) at 273 K was added to *N*-(4-tolyl)acetamide (1.49 g, 10 mmol), and heated at 353 K for 15 h. The mixture was then poured onto ice, and the white product was collected and dried. The compound was purified by recrystallization from a petroleum ether/ethyl acetate mixture.

### Refinement

Carbon-bound H-atoms were placed in calculated positions (C–H 0.93–0.96 Å) and were included in the refinement in the riding model approximation with *U*_iso_(H) set to 1.2–1.5*U*_eq_(C).

### Crystal data

**Table:**

C_11_H_8_ClNO	*F*(000) = 212
*M**_r_* = 205.63	*D*_x_ = 1.466 Mg m^−^^3^
Monoclinic, *P**c*	Mo *K*α radiation, λ = 0.71073 Å
Hall symbol: P -2yc	Cell parameters from 1352 reflections
*a* = 5.944 (1) Å	θ = 2.0–20.7°
*b* = 3.9210 (19) Å	µ = 0.37 mm^−^^1^
*c* = 20.390 (2) Å	*T* = 290 K
β = 101.377 (15)°	Block, colorless
*V* = 465.9 (2) Å^3^	0.25 × 0.15 × 0.15 mm
*Z* = 2	

### Data collection

**Table:**

Oxford Diffraction Excalibur diffractometer	2052 independent reflections
Radiation source: fine-focus sealed tube	1831 reflections with *I* > 2σ(*I*)
graphite	*R*_int_ = 0.028
ω scans	θ_max_ = 27.5°, θ_min_ = 3.5°
Absorption correction: multi-scan (*CrysAlis PRO*; Oxford Diffraction, 2009)	*h* = −7→7
*T*_min_ = 0.913, *T*_max_ = 0.947	*k* = −5→5
5980 measured reflections	*l* = −26→25

### Refinement

**Table:**

Refinement on *F*^2^	Secondary atom site location: difference Fourier map
Least-squares matrix: full	Hydrogen site location: inferred from neighbouring sites
*R*[*F*^2^ > 2σ(*F*^2^)] = 0.034	H-atom parameters constrained
*wR*(*F*^2^) = 0.089	*w* = 1/[σ^2^(*F*_o_^2^) + (0.0584*P*)^2^] where *P* = (*F*_o_^2^ + 2*F*_c_^2^)/3
*S* = 1.00	(Δ/σ)_max_ < 0.001
2052 reflections	Δρ_max_ = 0.19 e Å^−^^3^
128 parameters	Δρ_min_ = −0.17 e Å^−^^3^
2 restraints	Absolute structure: Flack (1983), 990 Friedel pairs
Primary atom site location: structure-invariant direct methods	Flack parameter: 0.02 (6)

### Fractional atomic coordinates and isotropic or equivalent isotropic displacement parameters (Å^2^)

**Table:**

	*x*	*y*	*z*	*U*_iso_*/*U*_eq_	
Cl1	1.00002 (8)	1.11653 (14)	0.50000 (3)	0.04862 (17)	
O1	0.4626 (4)	0.5444 (6)	0.55814 (8)	0.0676 (6)	
N1	0.7865 (3)	0.9885 (5)	0.37983 (9)	0.0382 (4)	
C1	0.7703 (3)	0.9492 (5)	0.44201 (10)	0.0351 (5)	
C2	0.5905 (3)	0.7858 (5)	0.46557 (10)	0.0340 (4)	
C3	0.4137 (4)	0.6644 (5)	0.41837 (10)	0.0341 (4)	
H3	0.2906	0.5561	0.4315	0.041*	
C4	0.4163 (3)	0.7021 (5)	0.34984 (10)	0.0320 (4)	
C5	0.2378 (4)	0.5831 (5)	0.29839 (10)	0.0366 (4)	
H5	0.1096	0.4792	0.3094	0.044*	
C6	0.2519 (4)	0.6196 (5)	0.23214 (10)	0.0383 (5)	
C7	0.4490 (4)	0.7786 (6)	0.21674 (10)	0.0454 (5)	
H7	0.4598	0.8019	0.1721	0.054*	
C8	0.6212 (4)	0.8971 (6)	0.26419 (11)	0.0441 (5)	
H8	0.7474	1.0015	0.2521	0.053*	
C9	0.6099 (4)	0.8627 (5)	0.33289 (10)	0.0335 (4)	
C10	0.5895 (4)	0.7365 (7)	0.53783 (11)	0.0470 (5)	
H10	0.6930	0.8615	0.5688	0.056*	
C11	0.0664 (4)	0.4932 (7)	0.17678 (10)	0.0501 (6)	
H11A	−0.0398	0.3562	0.1952	0.075*	
H11B	0.1332	0.3584	0.1463	0.075*	
H11C	−0.0131	0.6841	0.1534	0.075*	

### Atomic displacement parameters (Å^2^)

**Table:**

	*U*^11^	*U*^22^	*U*^33^	*U*^12^	*U*^13^	*U*^23^
Cl1	0.0366 (3)	0.0578 (3)	0.0488 (3)	−0.0066 (3)	0.00193 (19)	−0.0056 (3)
O1	0.0607 (12)	0.1037 (15)	0.0375 (9)	−0.0252 (12)	0.0074 (8)	0.0170 (10)
N1	0.0348 (9)	0.0388 (8)	0.0428 (10)	−0.0031 (7)	0.0118 (7)	−0.0002 (7)
C1	0.0309 (11)	0.0349 (11)	0.0389 (11)	0.0019 (8)	0.0056 (8)	−0.0021 (8)
C2	0.0325 (11)	0.0377 (10)	0.0325 (9)	0.0060 (9)	0.0083 (8)	0.0028 (8)
C3	0.0310 (10)	0.0375 (10)	0.0354 (11)	0.0007 (8)	0.0106 (8)	0.0031 (8)
C4	0.0327 (10)	0.0302 (10)	0.0337 (10)	0.0022 (8)	0.0077 (8)	0.0012 (8)
C5	0.0346 (11)	0.0376 (11)	0.0375 (10)	−0.0002 (8)	0.0067 (8)	0.0003 (8)
C6	0.0412 (12)	0.0384 (11)	0.0348 (10)	0.0030 (9)	0.0062 (9)	−0.0016 (8)
C7	0.0575 (15)	0.0519 (12)	0.0294 (10)	0.0032 (11)	0.0148 (10)	0.0014 (9)
C8	0.0480 (13)	0.0471 (12)	0.0421 (11)	−0.0048 (10)	0.0207 (10)	0.0018 (10)
C9	0.0355 (10)	0.0326 (10)	0.0337 (10)	0.0021 (8)	0.0099 (8)	0.0003 (8)
C10	0.0412 (13)	0.0631 (14)	0.0350 (10)	−0.0026 (11)	0.0035 (9)	0.0021 (11)
C11	0.0573 (15)	0.0555 (13)	0.0346 (11)	−0.0024 (12)	0.0019 (10)	−0.0041 (10)

### Geometric parameters (Å, °)

**Table:**

Cl1—C1	1.748 (2)	C5—H5	0.9300
O1—C10	1.196 (3)	C6—C7	1.416 (3)
N1—C1	1.300 (2)	C6—C11	1.498 (3)
N1—C9	1.365 (3)	C7—C8	1.345 (3)
C1—C2	1.409 (3)	C7—H7	0.9300
C2—C3	1.363 (3)	C8—C9	1.422 (3)
C2—C10	1.487 (3)	C8—H8	0.9300
C3—C4	1.408 (3)	C10—H10	0.9300
C3—H3	0.9300	C11—H11A	0.9600
C4—C9	1.414 (3)	C11—H11B	0.9600
C4—C5	1.416 (3)	C11—H11C	0.9600
C5—C6	1.377 (3)		
			
C1—N1—C9	116.50 (17)	C8—C7—C6	122.5 (2)
N1—C1—C2	126.42 (19)	C8—C7—H7	118.7
N1—C1—Cl1	114.64 (15)	C6—C7—H7	118.7
C2—C1—Cl1	118.93 (14)	C7—C8—C9	119.9 (2)
C3—C2—C1	116.68 (17)	C7—C8—H8	120.0
C3—C2—C10	120.06 (19)	C9—C8—H8	120.0
C1—C2—C10	123.25 (19)	N1—C9—C4	122.69 (17)
C2—C3—C4	120.41 (18)	N1—C9—C8	118.51 (19)
C2—C3—H3	119.8	C4—C9—C8	118.81 (19)
C4—C3—H3	119.8	O1—C10—C2	123.4 (2)
C3—C4—C9	117.27 (17)	O1—C10—H10	118.3
C3—C4—C5	123.20 (18)	C2—C10—H10	118.3
C9—C4—C5	119.53 (17)	C6—C11—H11A	109.5
C6—C5—C4	120.73 (19)	C6—C11—H11B	109.5
C6—C5—H5	119.6	H11A—C11—H11B	109.5
C4—C5—H5	119.6	C6—C11—H11C	109.5
C5—C6—C7	118.4 (2)	H11A—C11—H11C	109.5
C5—C6—C11	121.8 (2)	H11B—C11—H11C	109.5
C7—C6—C11	119.78 (19)		
			
C9—N1—C1—C2	1.0 (3)	C5—C6—C7—C8	−0.6 (3)
C9—N1—C1—Cl1	−179.69 (15)	C11—C6—C7—C8	−180.0 (2)
N1—C1—C2—C3	−1.7 (3)	C6—C7—C8—C9	0.4 (3)
Cl1—C1—C2—C3	179.00 (15)	C1—N1—C9—C4	0.9 (3)
N1—C1—C2—C10	177.0 (2)	C1—N1—C9—C8	−179.45 (19)
Cl1—C1—C2—C10	−2.3 (3)	C3—C4—C9—N1	−2.0 (3)
C1—C2—C3—C4	0.5 (3)	C5—C4—C9—N1	178.91 (17)
C10—C2—C3—C4	−178.30 (18)	C3—C4—C9—C8	178.38 (18)
C2—C3—C4—C9	1.2 (3)	C5—C4—C9—C8	−0.7 (3)
C2—C3—C4—C5	−179.73 (19)	C7—C8—C9—N1	−179.4 (2)
C3—C4—C5—C6	−178.44 (18)	C7—C8—C9—C4	0.2 (3)
C9—C4—C5—C6	0.6 (3)	C3—C2—C10—O1	13.5 (4)
C4—C5—C6—C7	0.0 (3)	C1—C2—C10—O1	−165.1 (3)
C4—C5—C6—C11	179.4 (2)		
